# Static and temporal dynamic changes of intrinsic brain activity in early-onset and adult-onset schizophrenia: a fMRI study of interaction effects

**DOI:** 10.3389/fneur.2024.1445599

**Published:** 2024-11-25

**Authors:** Jingli Chen, Yarui Wei, Kangkang Xue, Xinyu Gao, Mengzhe Zhang, Shaoqiang Han, Baohong Wen, Guangyu Wu, Jingliang Cheng

**Affiliations:** ^1^Department of Magnetic Resonance Imaging, The First Affiliated Hospital of Zhengzhou University, Zhengzhou, China; ^2^Laboratory for Functional Magnetic Resonance Imaging and Molecular Imaging of Henan Province, Zhengzhou, China; ^3^Department of Radiology, Renji Hospital, Shanghai Jiao Tong University School of Medicine, Shanghai, China

**Keywords:** amplitude of low-frequency fluctuation, dynamic intrinsic brain activity, compensatory effect, EOS, AOS

## Abstract

**Background:**

Schizophrenia is characterized by altered static and dynamic spontaneous brain activity. However, the conclusions regarding this are inconsistent. Evidence has revealed that this inconsistency could be due to mixed effects of age of onset.

**Methods:**

We enrolled 66/84 drug-naïve first-episode patients with early-onset/adult-onset schizophrenia (EOS/AOS) and matched normal controls (NCs) (46 adolescents, 73 adults), undergoing resting-state functional magnetic resonance imaging. Two-way ANOVA was used to determine the amplitude of low-frequency fluctuation (ALFF) and dynamic ALFF (dALFF) among the four groups.

**Result:**

Compared to NCs, EOS had a higher ALFF in inferior frontal gyrus bilateral triangular part (IFG-tri), left opercular part (IFG-oper), left orbital part (IFG-orb), and left middle frontal gyrus (MFG). The AOS had a lower ALFF in left IFG-tri, IFG-oper, and lower dALFF in left IFG-tri. Compared to AOS, EOS had a higher ALFF in the left IFG-orb, and MFG, and higher dALFF in IFG-tri. Adult NCs had higher ALFF and dALFF in the prefrontal cortex (PFC) than adolescent NCs. The main effects of diagnosis were found in the PFC, medial temporal structures, cerebrum, visual and sensorimotor networks, the main effects of age were found in the visual and motor networks of ALFF and PFC of dALFF.

**Conclusion:**

Our findings unveil the static and dynamic neural activity mechanisms involved in the interaction between disorder and age in schizophrenia. Our results underscore age-related abnormalities in the neural activity of the PFC, shedding new light on the neurobiological mechanisms underlying the development of schizophrenia. This insight may offer valuable perspectives for the specific treatment of EOS in clinical settings.

## 1 Introduction

The age of onset is an important defining characteristic of schizophrenia, and is associated with neurodevelopmental disorders (1). Early-onset schizophrenia (EOS: age of onset 13–18 years) has been reported to be more likely to be more severe, less responsive to treatment, and at greater risk of causing side effects than adult-onset schizophrenia (AOS: age of onset after18 years) (2, 3). Furthermore, considering that atypical neurodevelopment was believed to contribute to the etiology of schizophrenia, EOS patients offer a window for investigating the neurodevelopmental hypothesis of schizophrenia (4, 5). However, the neurobiological mechanism of this disorder remains elusive. Therefore, research on early-onset schizophrenia can provide a deeper understanding of the pathophysiological processes underlying this condition, which holds significant implications for early intervention and treatment.

Resting-state functional magnetic resonance imaging (rs-fMRI) offers the potential to understand the pathophysiology of schizophrenia by uncovering the neurobiological foundations of pathophysiological mechanisms that remain imperceptible at the behavioral level (6). There is growing evidence that the human brain’s low-frequency (between 0.01 and 0.1 Hz) spontaneous fluctuations are physiologically significant and are connected to spontaneous neural activities (7–9). A reduced amplitude of low-frequency fluctuation (ALFF) could indicate disease-related functional impairment, whereas an increased ALFF could be a sign of compensatory mechanisms to maintain normal cognition (10, 11). In individuals with schizophrenia, aberrant functional alterations in multiple cortical and subcortical brain regions are widely observed, reflecting disturbances in spontaneous brain activity (12, 13), in which the prefrontal cortex (PFC) governs attention to stimuli as well as information flow in and out of storage modules in its role as the central executive (14). Nonetheless, findings obtained from conventional functional neuroimaging have been grounded in the assumption that brain activity remains static throughout fMRI scans. However, this perspective can be considered overly simplistic in capturing the comprehensive intricacies of the human brain, which is an inherently dynamic and complex system (15).

Brain dynamics, defined by the temporal flexibility and diversity of brain processes, provide information on variations in the strength or spatial dynamic structures beyond static viewpoints (16). Moreover, previous research has demonstrated that the resting brain is a highly dynamic system with ever-changing mental states as time changes (17, 18). Recent study has associated the dynamic properties of brain activity with schizophrenia (19). Specifically, higher temporal dynamics in the brain regions involved in sensory processing are inversely associated with negative symptoms in EOS patients (20). The dynamic ALFF (dALFF) method, which illustrates temporal changes in energy consumption and reveals neural flexibility, is used to capture dynamic brain activity using a sliding window approach with greater sensitivity (6, 21, 22). Thus, both ALFF and dALFF methods may reflect brain and clinical characteristic (23) and provide complementary information about regional spontaneous brain activity (13). In addition, previous research on ALFF and dALFF has suggested that abnormalities in intrinsic brain activities may be a potential disease-related mechanism of schizophrenia and affect aspects of patients’ cognitive functions (16, 24, 25). Furthermore, the static and dynamic connections of EOS and AOS exhibit distinct neuroimaging findings, potentially aiding the differentiation between these conditions (10, 26, 27). Differences in the age of onset of schizophrenia may explain the conflicting findings and heterogeneity of this disorder (28). Shreds of evidence suggest that the spontaneous brain activity of schizophrenia is differently altered at different stages according to the mature state of brain development (29, 30). From the perspective of developmental trajectories, developmental maturation involves important changes in the control of various transmitter systems and their excitatory-inhibitory balance, which is particularly relevant to synchronized oscillations (31). Considering the developmental incompleteness of the implicated neural systems during adolescence, it is necessary to investigate the distinctive characteristics of cerebral dynamics within this cohort particularly. To the best of our knowledge, no research has used cross-sectional rs-fMRI data to investigate the neurobiological mechanisms driving EOS through the perspective of age-disorder interaction.

In this study, we used ALFF and dALFF methods to assess spontaneous brain activity disturbances. To determine whether and how an interaction exists between schizophrenia and age, four groups of participants (AOS/EOS and adolescent/adult normal controls (NCs)) were recruited. Based on prior research, we hypothesize that the interaction between schizophrenia and age may lead to various patterns of spontaneous neuronal activity, both static and dynamic. In addition, we hypothesized that group differences in ALFF and dALFF would be observed, and that these differences would explain some of the variations in clinical symptoms that distinguish EOS from AOS.

## 2 Methods

### 2.1 Participants

The first-episode patients with schizophrenia (84 with AOS and 66 with EOS, right-handed) were recruited from the outpatient services at the First Affiliated Hospital of Zhengzhou University to participate in an R-fMRI study. All patients met DSM-IV-TR criteria for schizophrenia and had no history of medication use. The positive and negative syndrome scale (PANSS) was used to evaluate the psychiatric symptoms of schizophrenia in all enrolled patients. The MATRICS Consensus Cognitive Battery (MCCB) was used to assess baseline cognitive function in 44 patients with EOS and 49 patients with AOS (Keith H (32)). It involves seven cognitive areas:(1) Speed of Processing Information (Speed of Processing Information, SOP), (2) Attention and Vigilance Awareness (Continuous Performance Test: Identical Pairs, CPT-IT), (3) Working Memory (Wechsler Memory Scale, 3rd ed., WMS-III), (4) Verbal Learning (Hopkins Verbal Learning Test, HVLT), (5) Visual Learning (Brief Visuospatial Memory Test, BVMT), (6) Reasoning and Problem-Solving (Neuropsychological Assessment Battery, NAB), and (7) Social Cognition (Mayer-Salovey-Caruso Emotional Intelligence Test, MSCEIT). The MCCB scoring program generates T-scores that are standardized and corrected for age and sex. Patients were excluded if they had been diagnosed with other medical conditions, such as severe medical illnesses, physical impairments, or coexisting serious medical conditions such as mental retardation, dementia, and severe cognitive dysfunction. Additionally, individuals with substance abuse issues, such as alcohol dependence, and those participating in other clinical studies were excluded.

Furthermore, 119 NCs (73 adults and 46 adolescents, all right-handed) were recruited locally and underwent uniform screening. None had a history of medical, psychiatric disorders, or substance abuse issues. Participants provided informed consent, and the study protocol reviewed and approved by the Ethics Committee of the First Affiliated Hospital of Zhengzhou University.

### 2.2 Data acquisition

All participants underwent scans using a 3.0 T MRI scanner with an 8-channel receiver array head coil (Discovery MR750, GE, United States). Foam padding and earplugs were used to limit head movements and scanner noise. All participants’ head movement parameters were less than 2.5 mm displacement and 2.5° of rotation. With their eyes closed, all participants were encouraged to remain awake. The rs-fMRI images were collected using the following parameters in a gradient-echo single-shot echo-planar imaging (GRE-SS-EPI) sequence: repetition time/echo time = 2000/30 ms, slice thickness = 4 mm, slice gap = 0.5 mm, flip angle = 90°, slice number = 32, field of view = 22 × 22 cm^2^, number of averages = 1, matrix size = 64 × 64, and voxel size = 3.4375 × 3.4375 × 3.4375 mm^3^. The duration of the resting state scan was 6 min.

### 2.3 Rs-MRI data processing

Data was processed using the Data Processing Assistant for Resting-State fMRI Analysis Toolkit (DPARSF V4.3), SPM12, and customized MATLAB pipeline. The following steps were performed: (1) the first five volumes were removed to allow for signal stabilization, (2) slice timing correction to the middle slice was carried out, (3) the images were realigned to exclude participants with maximum head motion greater than 2.5 mm or rotation greater than 2.5°, (4) the rs-fMRI data was normalized using EPI templates (resampled voxel size = 3 × 3 × 3 mm^3^), (5) detrending was performed, (6) A filter ranging from 0.01 to 0.1 Hz was employed to eliminate low-frequency drifts and high-frequency physiological noise, (7) scrubbing was performed with cubic spline interpolation to remove the “bad” time points and their 1-back and 2-after time points based on a framewise displacement (FD) threshold of 0.5 mm, (8) regression of the Friston-24 motion parameters, and white matter and cerebrospinal fluid signals, (9) all data were smoothed with a 6 mm isotropic Gaussian kernel for statistical analyses.

### 2.4 Estimation of the ALFF and dALFF

The ALFF was calculated using DPABI software toolbox. The power spectrum was produced after transforming each voxel’s time series to the frequency domain using a fast Fourier transform. Since the power of a given frequency was proportional to the square of amplitude of this frequency component, the square root was calculated at each frequency of the power spectrum, and the averaged square root was obtained across 0.01–0.1 Hz at each voxel (33). The averaged square root was used as ALFF value. The dALFF was calculated using Dynamic Brain Connectome toolbox. Based on the processed data, the dALFF calculation was restricted to the gray matter mask. The sliding window method was used to characterize dynamic neural activity. A series of ALFF maps were produced for every participant under various sliding windows. Subsequently, we calculated the variance of these plots using the standard deviation (SD) to evaluate the temporal variability of dALFF (dALFF variability) (34). Finally, by subtracting the global values’ mean and dividing the result by the SD, the dALFF variability per voxel for every participant was further transformed into z-scores. The “rule of thumb” dictates that the minimum window length should not fall below 1/f min (the cut-off frequency), as shorter time segments can introduce spurious fluctuations. Following this principle, f min was identified as the minimum frequency within the time series (35). To optimize the trade-off between capturing rapidly evolving dynamic interactions (facilitated by shorter windows) and attaining precise estimations of inter-regional correlations (enabled by longer windows), a sliding-window technique with a final duration of 50 TRs (100 s) was implemented (36), with a window overlap of 60% was chosen (window shifted by 20 TRs) (37). We also tested other sliding-window durations (30 TR and 80 TR) to investigate their potential influence on dALFF results.

### 2.5 Statistical analysis

The chi-square (*χ*^2^) test for sex was used to assess demographic and clinical data. For additional demographic parameters and clinical scores, two-sample t-tests or two-sample Mann–Whitney U tests were used. Differences were considered statistically significant at *p <* 0.05. For ALFF and dALFF comparisons, a two-way analysis of variance (ANOVA) was used to examine the main effects of the diagnosis (schizophrenia vs. controls) and age (adolescent vs. adult) and their interaction effects. Sex, educational level, and mean FD were entered as nuisance covariates. Multiple comparisons were corrected according to Gaussian random field (GRF) theory (voxel-wise *p* < 0.001, cluster-wise *p* < 0.05, two-tailed, and cluster extent threshold at k > 20) (38). From corrected statistical maps of the main effects of diagnosis and age as well as interaction effects, we extracted the average dALFF variance and ALFF values of all voxels for each cluster, performed post-hoc comparisons using Mann–Whitney U test, and corrected for multiple comparisons (*p* < 0.05/2 for main effect analyses, *p* < 0.05/4 for interaction effect analyses, Bonferroni-corrected). Furthermore, a correlation analysis between clinical measures (PANSS and, MCCB) and significant outcomes between the groups was performed using Spearman’s rank correlation (Bonferroni-corrected).

## 3 Results

### 3.1 Demographic and clinical data

Statistically differences were found regarding education (years) (*p* < 0.001; *p* < 0.001), age between adults and adolescents (*p* < 0.001) and NAB sub-scores (*p* = 0.040). However, no statistical differences were observed regarding the other clinical indicators (Table 1).

**Table 1:** Demographic and clinical data of adolescents and adults between schizophrenia patients and NCs.

Demographics	Patients (150)		Normal controls (119)		Comparison	
	Adults (84)	Adolescents (66)	Adults (73)	Adolescents (46)	Patients vs. controls	Adults vs. Adolescents
Sex (male/female)	45/39	30/36	34/39	27/19	*χ*^2a^ = 0.04(*p* = 0.84)	*χ*^2a^ = 0.01(*p* = 0.93)
Age (year)	22.7 ± 4.1	15.5 ± 1.3	22.4 ± 1.6	15.2 ± 1.6	Z^b^= − 0.96(*p* = 0.34)	**Z**^**b**^ **=14.0(*p* = 0.00)**
Education(year)	12.3 ± 2.7	9.5 ± 1.4	16.2 ± 1.4	10.0 ± 2.0	**Z** ^**b**^ **= − 6.45(*p* = 0.00)**	**Z** ^**b**^ **=10.9(*p* = 0.00)**
Duration of illness(months)	12.4 ± 13.0	12.1 ± 12.5	–	–	Z^c^= − 1.60(*p* = 0.11)	–
PNASS	N = 84	N = 66	–	–	–	–
Total scores	87.5 ± 18.4	83.0 ± 16.7	–	–	–	t^c^=1.53, *p* = 0.128
Positive	20.6 ± 5.5	19.7 ± 6.5	–	–	–	Z^b^= − 0.80, *p* = 0.43
Negative	22.8 ± 6.8	22.0 ± 6.0	–	–	–	t^c^=0.77, *p* = 0.44
General	44.1 ± 9.2	41.2 ± 9.2	-	-	–	Z^b^= − 1.30, *p* = 0.20
Mean FD	0.1 ± 0.1	0.1 ± 0.1	–	–	–	Z^b^= − 0.91, *p* = 0.37
MCCB	N = 49	N = 46	–	–	–	–
SOP	26.9 ± 12.5	28.4 ± 12.5	–	–	–	t^c^= − 0.59, *p* = 0.56
CPT_IP	30.8 ± 11.5	33.2 ± 16.2	–	–	–	t^c^= − 0.82, *p* = 0.42
WMS-III	39.7 ± 10.1	40.8 ± 12.9	–	–	–	t^c^= − 0.43, *p* = 0.67
HVLT_R	36.8 ± 9.7	40.8 ± 11.1	–	–	–	t^c^= − 1.85, *p* = 0.07
BVMT_R	35.7 ± 17.1	42.3 ± 12.8	–	–	–	t^c^= − 1.85, p = 0.07
NAB	33.0 ± 9.1	33.7 ± 10.2	–	–	–	**t** ^**c**^ **= − 2.08, *p* = 0.04**
MSCEIT	37.6 ± 14.0	37.9 ± 16.2	–	–	–	t^c^= − 0.07, *p* = 0.94

Values are mean ± SEM. Bold values indicate statistically significant differences. PNASS, positive and negative syndrome scale; MCCB: MATRICS Consensus Cognitive Battery; FD, framewise displacement. ^a^The *χ*^2^ value for gender distribution was obtained by chi-square test. ^b^The *Z* values were obtained by the Mann–Whitney test. ^c^The *t* values were obtained by the two-sample *t*-test. SOP, Speed of Processing Information; CPT-IT, Continuous Performance Test, Identical Pairs; WMS-III, Wechsler Memory Scale, 3rd ed; HVLT, Hopkins Verbal Learning Test; BVMT, Brief Visuospatial Memory Test; NAB, Neuropsychological Assessment Battery; MSCEIT, Mayer-Salovey-Caruso Emotional Intelligence Test.

### 3.2 Main effects

**Figure 1:**
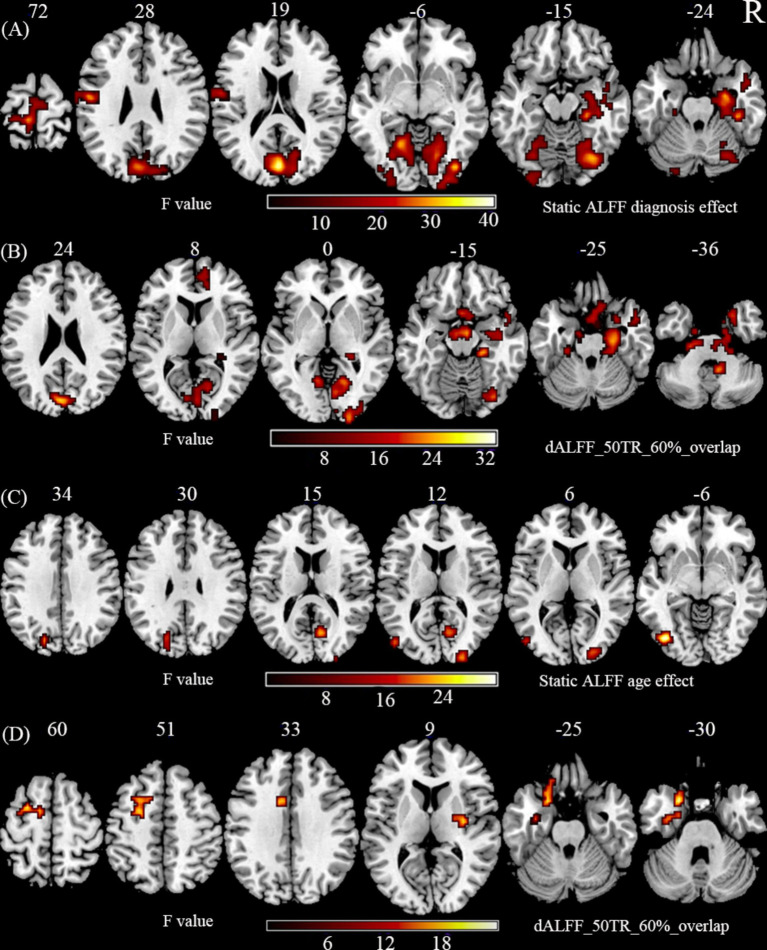
The main effect of diagnosis and age. (A) Static ALFF shows the significant main effect of diagnosis in using two-way ANOVA. (B) dALFF with 50 TR and 60% overlap shows the significant main effect of diagnosis in using two-way ANOVA. (C) Static ALFF shows the significant main effect of age in using two-way ANOVA. (D) dALFF with 50 TR and 60% overlap shows the significant main effect of age in using two-way ANOVA. The statistical significance level in **(**A–D) was set at *p* < 0.001 (two-tail) and cluster size at *p* < 0.05, GRF corrected.

Regarding the main effects of diagnosis (Figures 1A,B; Supplementary Table S1), we found abnormal ALFF and dALFF in right parahippocampal gyrus, right hippocampus, right middle temporal lobe (MTG), bilateral calcarine gyrus, bilateral cuneus, bilateral lingual gyrus, right fusiform gyrus, right inferior occipital gyrus (IOG), right middle occipital gyrus (MOG), and right lobule IX. We also discovered an abnormal ALFF in left frontal lobe, left fusiform gyrus, left MOG, right superior occipital gyrus (SOG), left lobule IV/V, right lobule VI, left precuneus, left postcentral gyrus, and left paracentral gyrus. An abnormal dALFF was observed in right superior temporal lobe (STG), right medial frontal lobe, right anterior cingulum, right lobule VII, right lobule X, and left lobule IX. The planned post-hoc analysis revealed that schizophrenia was associated with increased ALFF and dALFF in regions with high cognitive function associated with the limbic network and auditory network regions and decreased ALFF and dALFF in the low-level brain areas associated with visual and sensorimotor regions, irrespective of age (Supplementary Tables S2, S3).

Regarding the main effects of age (Figures 1C,D; Supplementary Table S4), we found an abnormal ALFF in the right calcarine gyrus, left IOG, bilateral MOG, and right paracentral gyrus. We found an abnormal dALFF in left fusiform gyrus, right pallidum, left MTG, and left frontal lobe [SFG, STG, inferior frontal gyrus (IFG), IFG-orb]. The planned post-hoc analysis revealed that adult NCs were associated with a decreased ALFF in the lower brain regions related to visual, and motor functions and an increased dALFF in the PFC compared to adolescent NCs, irrespective of the disorder status (Supplementary Tables S5, S6).

### 3.3 Interaction effects

**Figure 2:**
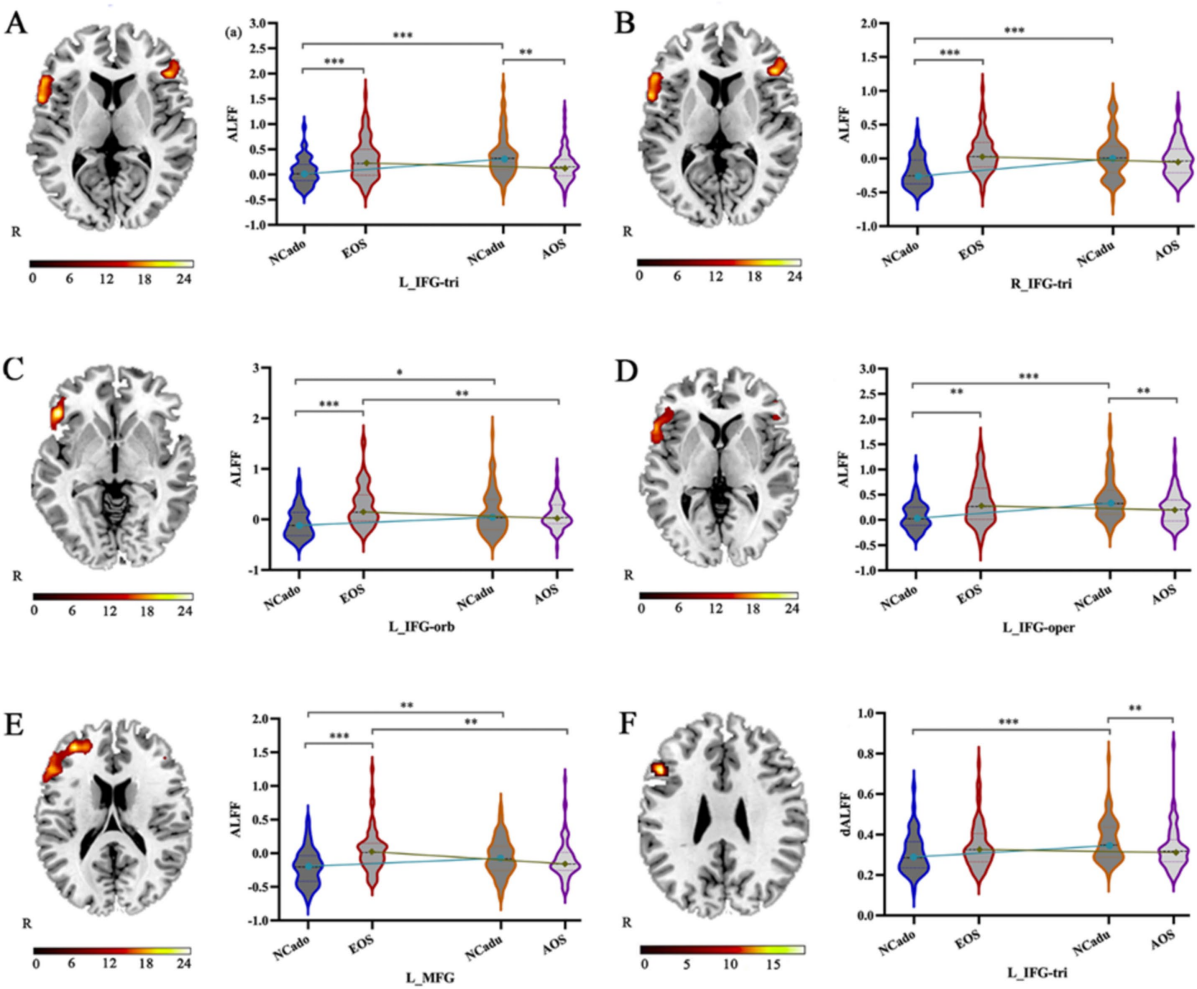
The interaction effect of age and ALFF and dALFF alterations caused by diagnosis. (A–F) The significant interaction effect in the left IFG-tri, right IFG-tri, left IFG-oper, left IFG-orb, and left MTG is shown by static ALFF and dynamic ALFF with 50 TR and 60% overlap using two-way ANOVA (right) and planned post-hoc analysis (left). IFG-tri: triangular part of the inferior frontal gyrus; IFG-oper: opercular part of the inferior frontal gyrus; IFG-orb: orbital part of the inferior frontal gyrus; MFG: middle frontal gyrus. NCadu: adult normal controls; NCado: adolescent normal controls. *p < 0.05; **p < 0.01; ***p < 0.001.

Significant interaction effects (Figure 2; Table 2) were observed in the left IFG-tri in ALFF and dALFF. The ALFF method also included the right IFG-tri, left IFG-oper, left IFG-orb, and left middle frontal gyrus (MFG). The planned post-hoc analysis revealed the following results:(1) Compared to adult NCs, AOS patients had lower ALFFs in the left IFG-tri (*Z* = −3.231, *p* = 0.001), IFG-oper (*Z* = −3.037, *p* = 0.002), and lower dALFFs in the left IFG-tri (*Z* = −2.661, *p* = 0.008), (2) Compared to adolescent NCs, EOS patients had higher ALFFs in bilateral IFG-tri (left, *Z* = −3.448, *p* = 0.001; right, *Z* = −4.903, *p* < 0.001), left IFG-oper (*Z* = −2.634, *p* = 0.008), left IFG-orb (*Z* = −4.595, *p* < 0.001), and left MFG (*Z* = −4.323, *p* < 0.001), and higher dALFFs in left IFG-tri (*Z* = −4.323, *p* < 0.001), (3) Compared to AOS patients, those with EOS had higher ALFFs in the left IFG-orb (*Z* = −2.681, *p* = 0.007), left MFG (*Z* = −3.398, *p* = 0.001) and higher dALFFs in the IFG-tri (*Z* = −2.734, *p* = 0.006), (4) Compared to adolescent NCs, adult NCs had higher ALFFs in the bilateral IFG-tri (left, *Z* = −4.600, *p* < 0.001; right, *Z* = −4.022, *p* < 0.001), left IFG-oper (*Z* = −4.770, *p* < 0.001), left IFG-orb (*Z* = −2.527, *p* = 0.012), left MFG (*Z* = −2.892, *p* = 0.004) and higher dALFFs in the left IFG-tri (*Z* = −3.383, *p* = 0.001).

**Table 2:** Brain Regions with Significant Group Differences in the Interaction Effect.

Group differences	Regions	Cluster size (voxels)	Peak MNI coordinate			Peak *F* values
			X	Y	Z	
ALFF	L_ IFG-tri	129	−57	18	6	21.88
	R_ IFG-tri	43	48	30	9	23.14
	L_ IFG-oper	32	−57	15	6	21.77
	L_IFG-orb	38	−48	30	−3	26.00
	L_MFG	87	−33	45	15	24.10
dALFF	L_ IFG-tri	39	−48	21	28	18.41

L, left; R, right; MNI, Montreal Neurological Institute; IFG-tri, triangular part of the inferior frontal gyrus; IFG-oper, opercular part of the inferior frontal gyrus; IFG-orb, orbital part of the inferior frontal gyrus; MFG, middle frontal gyrus.

### 3.4 Correlation analyses

**Figure 3:**
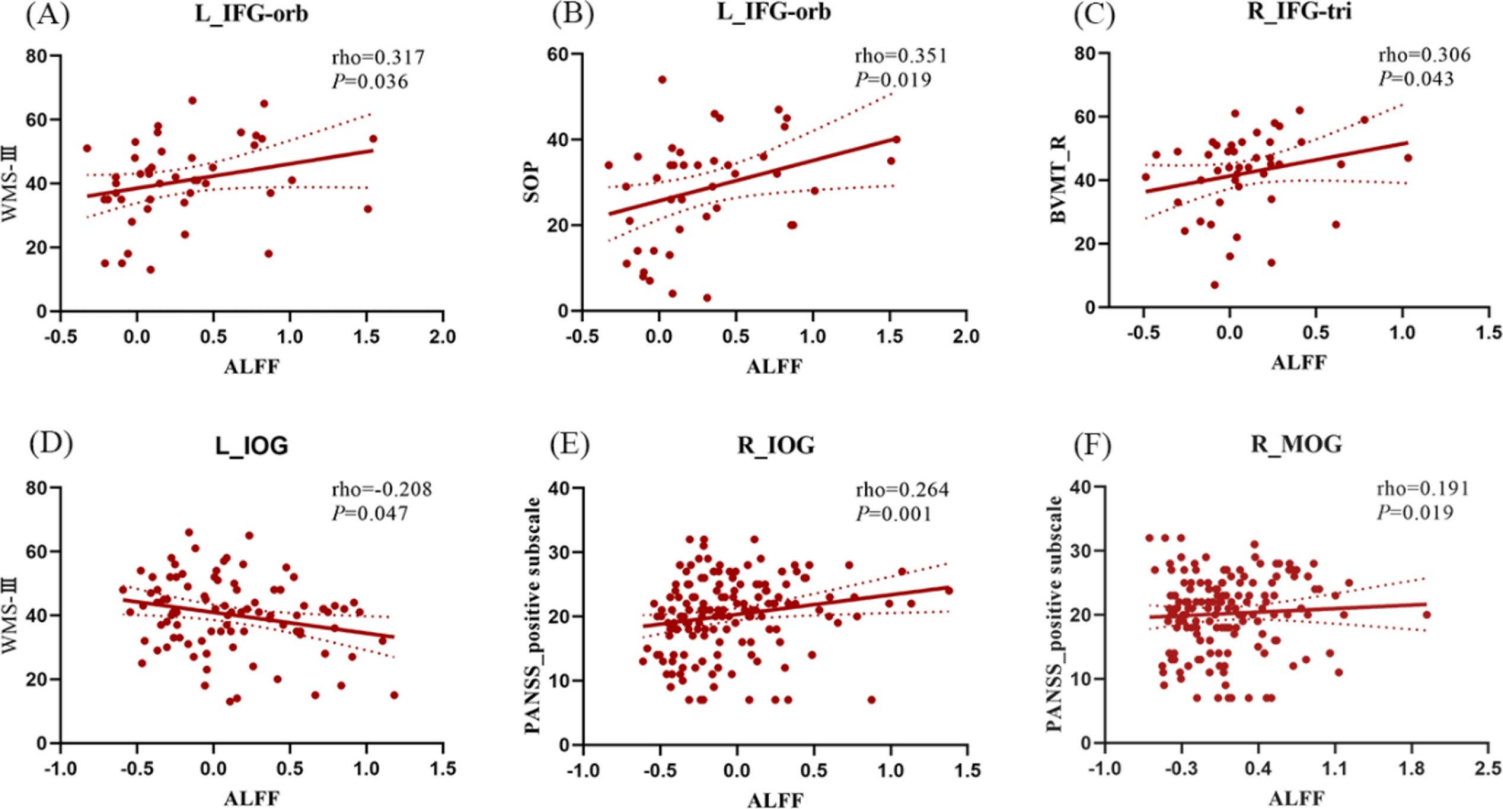
Correlation analysis between ALFF/dALFF changes caused by interaction effects and PANSS and MCCB scores in schizophrenia. (A,B) In EOS, ALFF of L_IFG-orb was positively correlated with WMS-III and SOP subscores; (C) In EOS, ALFF of R_IGF-tri was positively correlated with BVMT-R subscore; (D) In schizophrenia, ALFF of L_IOG was negtively correlated with WMS-III subscore. (E) In schizophrenia, the abnormal ALFF of the R_IOG was positively correlated with PANSS positive subscore; (F) In schizophrenia, the abnormal ALFF of the R_MOG was positively correlated with PANSS positive sub-score. L, left; R, right; IFG-orb, orbital part of the inferior frontal gyrus; IFG-tri, triangular part of the inferior frontal gyrus; IOG, inferior occipital gyrus; MOG, middle occipital gyrus. NCadu, adult normal controls; NCado, adolescent normal controls.

Regarding the interaction effects, we found a positive correlation between the WMS-III, SOP sub-score and an increased ALFF of left IFG-orb (rho = 0.317, *p* = 0.036; rho = 0.351, *p* = 0.019), a positive correlation between BVMT-R sub-score and an increased ALFF of right IFG-tri (rho = 0.306, *p* = 0.043) in EOS group (Figures 3A–C). In schizophrenia patients, the decreased ALFF of left IOG was negatively correlated with the WMS-III (rho = −0.208; *p* = 0.047) (Figure 3D). In addition, the decreased ALFFs of the right IOG (rho = 0.264; *p* = 0.001), MOG (rho = 0.191; *p* = 0.019) were positively correlated with the positive sub-scale of PANSS (Figures 3E,F). Multiple comparisons were adjusted using the Bonferroni method.

### 3.5 Validation analyses

In the dALFF with 30 TRs and a 60% overlap, 50 TRs and an 80% overlap, the anomalous interaction effect was mainly observed in the left IFG-tri. In the dALFF with 80 TRs and a 60% overlap, interaction results did not pass the correction (Supplementary Figure S1, S2; Supplementary Table S7). In the dALFF with 30 TRs and a 60% overlap, 50 TRs and an 80% overlap, and 80 TRs and a 60% overlap, most of the abnormal brain regions for the main effect of diagnosis were preserved, including the medial temporal network, auditory network, visual network, and cerebellar network (Supplementary Figures S2A–C; Supplementary Table S8). In dALFF with 30 TRs and a 60% overlap, 50 TRs and an 80% overlap, the abnormal brain areas for the main effect of age were observed mainly in the visual network, basal ganglia area, frontal lobe, auditory network, and medial temporal lobe network (Supplementary Figures S2D,E; Supplementary Table S9).

## 4 Discussion

This study used a 2 × 2 factorial design for diagnosis and age status. We identified static and dynamic intrinsic brain activities among four groups of participants using ALFF and dALFF methods, which provided complementary information regarding regional spontaneous brain activities. Our study found that schizophrenia patients have extensive ALFF or dALFF abnormalities in the brain, particularly in the PFC, medial temporal cortex, visual cortex, and cerebellar cortex regardless of age. Additionally, the development of cognitive function with age was associated with a gradual shift from primary visuospatial or motor system activation to higher cognitive system. More importantly, the interaction effects were mainly located in the PFC. There was a tendency for lower brain neural activity in AOS patients and higher brain neural activity in EOS patients, followed by higher PFC brain neural activity in normal adults. These findings may support the alteration of the PFC in EOS patients in terms of information transmission efficiency. However, this group of patients shows some compensation for cognitive deficits due to the influence of developmental processes.

### 4.1 Interaction effects

The PFC, which is divided into anatomical subregions, plays a key role in integrating incoming information and providing ‘top-down’ processing to coordinate behavior because of its extensive interconnections with sensory, motor, and subcortical regions, forming distinct networks responsible for various functions (39, 40). The regions comprising the IFG-oper, IFG-orb, and IFG-tri are anatomically integral components of the IFG and are widely acknowledged for their distinct roles in language processing, execution, and cognitive functions (41). Moreover, researches have demonstrated that the IFG-orb plays a pivotal role in semantic processing (42), and aberrations in the gray matter volume (GMV) of the IFG-orb may be associated with cognitive deficits in patients with first-episode schizophrenia (41). In addition, Iwashiro et al. reported that structural abnormalities in the IFG-tri are significantly associated with the occurrence of positive symptoms, and that this region may represent a developmental vulnerability in schizophrenia, whereas structural abnormalities in the IFG-oper may reflect disorder progression after the onset of schizophrenia (43). Unlike the IFG, the MFG, a major component of the dorsolateral prefrontal cortex, is associated with processing problems of higher abstraction, complexity, and decision-making (39, 40). As previously reported, age-related increases in PFC neural activity during normal development are associated with the enhanced maturation of higher-order cognitive functions (44, 45). Our study findings consistently demonstrated that static and dynamic neural activities in PFC regions, were consistently higher in adult NCs than adolescent NCs, possibly supporting the stated conclusion.

In contrast, in psychiatric conditions such as schizophrenia, age-related developmental processes exert a discernible influence on the activation state of the PFC, consequently affecting the maturation of cognitive function during adolescence (46, 47). In most of studies involving schizophrenia, adult patients typically exhibit diminished activity within the PFC (48), whereas the PFC activity in adolescent patients tends to be less constrained and more variable (49). Neural inhibition is important for synchronizing oscillatory activity (50), managing precise spike timing, and increasing neuronal transmission efficiency (51). Conversely, the abnormalities in cortical inhibitory activity of the PFC may disrupt effective communication between neuronal groups, indirectly responding to cognitively inefficient neural circuits. Previous studies have indicated that higher neural activity in left IFG is consistent with the neural inefficiency hypothesis in people who are most at risk for EOS (52) and serves as a compensatory mechanism for maintaining normal performance in EOS patients (53). Left IFG deficiencies are linked to inhibitory control, language, and internal conflict resolution (40), and right IFG deficiencies are linked to verbal/visuospatial working memory (WM) (54). David et al. observed that abnormalities in inhibitory neurons may lead to relative hyperactivity in EOS patients (David W (55).). Therefore, the relative hyperactivity of the PFC may reflect inefficient neural circuitry and may be interpreted as functional compensation to mobilize more cognitive efforts in EOS patients (56, 57). In our correlational analysis, the BVMT_R sub-scores exhibited a gradual increase in conjunction with elevated ALFF values in the right IFG-tri, whereas WMS-III and SOP sub-scores also increased with rising ALFF values in the left IFG-orb (Figures 3A–C). This might provide an indirect confirmation of the previous observation. Taken together, the increased ALFF and dALFF of the bilateral IFG-tri, left IFG-oper, left IFG-orb, left MFG, and left IFG-tri may indicate that EOS patients tend to exhibit compensatory effects, with the frontal cognitive control exhibiting inefficiency when disturbed.

### 4.2 Main effects of diagnosis

Our findings suggest that schizophrenia increases static or dynamic spontaneous brain activity in the medial temporal network, medial PFC, and posterior cerebellar lobules, whereas it decreases in the sensorimotor network, visual network, motor network, and anterior cerebellar lobules, regardless of age, which is consistent with previous studies. Abnormalities in these brain regions are widely associated with deficits in processing speed, verbal learning, problem-solving, visual learning, and attention/vigilance in schizophrenia (58, 59). Evidence suggests that anomalies in the fronto-limbic system, including the PFC, hippocampus, and parahippocampal gyrus, may play a role in the neuropsychological symptoms of the disorder (60). Increased ALFF and dALFF in the hippocampus, parahippocampus, and MTG may be related to common symptoms of schizophrenia, such as auditory verbal hallucinations, because these regions are associated with internally directed thinking and confusion based on their origins (61, 62). Additionally, this study identified abnormal ALFF and dALFF in several brain regions related to language function in early-stage schizophrenia patients (e.g., Broca’s area and fronto-insular cortex) (63, 64), partially supporting Crow’s language hypothesis of schizophrenia (65).

Furthermore, reduced activity in the primary cortex, including visual and somatosensory cortices, is a distinguishing trait of schizophrenia (8), and specific visual perception difficulties in schizophrenia have been thoroughly reported (66). A study on visual hallucinations found lower ALFFs in the occipital cortex and higher ALFFs in the visual association cortex, as well as altered cortico-subcortical functional connectivity, which supports the theory that visual hallucinations are controlled by “bottom-up” peripheral sensory input from the primary visual cortex and unconstrained “top-down” cognition in frontal areas (67). Neuroanatomical deficits in the ventral pathways and PFC may induce disintegration of the occipito-temporo-frontal circuitry and dysfunction, which may underlie the disturbances of visuospatial WM in schizophrenia (68). In addition, these results are consistent with brain atrophy trajectories in schizophrenia revealed by recent studies using novel data-driven approaches (such as disease progression modeling and epicenter mapping) (69–71). We also discovered that a decreased ALFFs in left IOG had a negative effect on the WMS-III score of the MCCB in schizophrenia (Figure 3D), and the right IOG, MOG, which was positively associated with positive sub-scores of the PANSS (Figures 3E,F). These findings suggest that the abnormal neural activity in the visual cortex of schizophrenia is associated with positive symptoms and cognitive performance. The somatosensory cortex is connected to many cortical and subcortical areas, allowing it to perform various of functions such as body representations, sensorimotor integration, and emotion regulation (72). In schizophrenia, abnormalities in somatosensory, auditory, or visual-evoked potentials/evoked fields have been found, implying that all sensory cortices are somewhat affected (73, 74). A reduced ALFF in the bilateral postcentral gyrus and paracentral lobule may be associated with cognitive dysfunction in schizophrenia (6).The cerebellum is now thought to be involved in cognitive and emotional activities in addition to motor functions (75). The anterior cerebellum and lobule VII form a network with the primary motor cortex (76), whereas the majority of posterior cerebellum is part of the networks linked with cognitive activities (77). Lobules VIII and IX are closely connected to the default mode network (78), which has been implicated in self-referential and stream-of-consciousness processing in schizophrenia (79). The decreased ALFFs or dALFFs in lobules IV/V and VI and increased ALFFs or dALFFs in lobules VIII, IX, and X, may imply compromised motor and cognitive functions in schizophrenia in our findings. These results support the widespread abnormalities in static or dynamic spontaneous brain activities in schizophrenia and their relationship with various aspects of cognitive impairment.

### 4.3 Main effect of age

Late adolescence and early adulthood are times of active neuronal growth in higher-order integrative cortices, such as STG and PFC, which may explain age-related improvements in cognitive functions (46). Furthermore, there is evidence that the development of cognitive functions is associated with a shift from visuospatial or motor system activation to higher cognitive system activation with increasing age (46, 80). While age-related decreases in responses are signs that a particular circuitry is no longer required, age-related increases in activation are regarded as increased maturity in reaching performance-enhancing regions (46, 80). Based on this perspective, we suggest that the dALFFs in the left SFG, IFG, and STG increase with age, whereas the ALFFs in the occipital lobe, dALFFs in the left fusiform gyrus and ALFF in right paracentral gyrus decrease, which is generally linked to the maturation of strategic processing and becomes less dependent on visual analysis and sensorimotor processing.

## 5 Limitations

This study had several limitations. Firstly, longitudinal studies are the most effective in investigating the relationship between age and schizophrenia. Therefore, longitudinal data analyses would be required future research regarding this subject matter. Secondly, the demographics were slightly less evenly distributed. The adolescent NCs had a relatively small number of participants compared to the adult NCs. Thirdly, the lack of cognitive scales in NCs group and the relatively small number of clinical cognitive scales collected in the patient groups may have limited our ability to detect correlations. Lastly, it is important to note that the duration of untreated psychosis variable was not included in our analysis, which could potentially impact the interpretation of our findings.

## 6 Conclusion

Overall, our findings may elucidate the interaction between schizophrenia and age in terms of neurobiological mechanisms. Specifically, the presence of reversed activation patterns in the PFC of patients with EOS and AOS suggests that those with EOS may exhibit more compensatory regulations to address the functional impairments that occur during the disorder course. Our findings also suggest that adolescent brain development exhibits greater plasticity or flexibility compared to adults in the later stages of the disease, offering unique opportunities for intervention. Finally, these combined static and dynamic patterns may serve as reliable indications in clinical studies and play complementary roles.

## Data Availability

The raw data supporting the conclusions of this article will be made available by the authors, without undue reservation.
