# Supplementary material for: Static and temporal dynamic changes of intrinsic brain activity in early-onset and adult-onset schizophrenia: a fMRI study of interaction effects
Source: Front Neurol. 2024 Nov 25;15:1445599. doi: 10.3389/fneur.2024.1445599 (PMC11625647; doi:10.3389/fneur.2024.1445599)
Supplement: Supplementary file 1 [file Table_1.docx]

Supplementary Material

# Supplementary Figures and Tables

**Table S1.** Brain Regions with Significant Group Differences in The Main Effect of Diagnosis

| Group differences | Regions | Cluster size  (voxels) | Peak MNI coordinate | | | Peak F  values |
| --- | --- | --- | --- | --- | --- | --- |
|  |  |  | X | Y | Z |  |
| *ALFF* | R_ ParaHippocampal | 120 | 26 | -13 | -24 | 22.76 |
|  | R_ Hippocampus | 81 | 27 | -9 | -21 | 31.87 |
|  | R_MTG | 31 | 48 | 6 | -27 | 22.05 |
|  | L_Frontal Lobe | 137 |  |  |  |  |
|  | R_ Calcarine | 273 | 14 | -62 | 6 | 23.57 |
|  | L_ Calcarine | 198 | -8 | -77 | 10 | 26.63 |
|  | R_IOG | 141 | 48 | -66 | -6 | 21.31 |
|  | L_IOG | 41 | -27 | -96 | -12 | 23.58 |
|  | R_MOG | 35 | 36 | -93 | 1 | 19.44 |
|  | L_MOG | 53 | -48 | -75 | 9 | 21.01 |
|  | R_SOG | 32 | 22 | -83 | 29 | 16.95 |
|  | R_ Fusiform | 109 | 27 | -30 | -15 | 26.03 |
|  | L_ Fusiform | 59 | -27 | -69 | -12 | 27.64 |
|  | R_Lingual | 282 | 19 | -68 | 0 | 35.82 |
|  | L_Lingual | 229 | -12 | -61 | 0 | 36.64 |
|  | L_Cuneus | 173 | 1 | -80 | 22 | 41.20 |
|  | R_Cuneus | 164 | 3 | -81 | 21 | 41.27 |
|  | R_ lobules Ⅸ | 31 | 15 | -41 | -43 | 22.47 |
|  | L_ lobules Ⅵ/Ⅴ | 21 | -17 | -54 | -11 | 16.06 |
|  | R_ lobules Ⅵ | 90 | 30 | -70 | -18 | 31.45 |
|  | L_ Precuneus | 129 | -9 | -43 | 71 | 15.05 |
|  | L_ Paracentral gyrus | 106 | -9 | -33 | 72 | 29.28 |
|  | L_ postcentral gyrus | 146 | -54 | -9 | 27 | 30.04 |
| *dALFF* | R_ ParaHippocampal | 114 | 20 | -30 | -9 | 28.09 |
|  | R_ Hippocampus | 65 | 29 | -12 | -20 | 19.31 |
|  | R_STG_Pole | 41 | 48 | 12 | -20 | 19.28 |
|  | R_MTG_Pole | 28 | 47 | 13 | -21 | 19.28 |
|  | R_Medial Frontal gyrus | 34 | 12 | 57 | 12 | 18.08 |
|  | R_ACC | 21 | 47 | 13 | -21 | 19.28 |
|  | R_Lingual | 101 | 18 | -63 | 0 | 21.90 |
|  | L_Lingual | 23 | -10 | -61 | 1 | 18.62 |
|  | R_ Calcarine | 90 | 15 | -66 | 7 | 16.62 |
|  | L_ Calcarine | 55 | -2 | -77 | 17 | 18.49 |
|  | L_Cuneus | 78 | 0 | -81 | 21 | 27.11 |
|  | R_Cuneus | 30 | 3 | -83 | 24 | 21.92 |
|  | R_IOG | 77 | 24 | -99 | -3 | 22.74 |
|  | R_MOG | 21 | 36 | -91 | 1 | 12.85 |
|  | R_ Fusiform | 27 | 23 | -30 | -16 | 25.07 |
|  | R_ lobules Ⅷ | 28 | 19 | -39 | -50 | 19.06 |
|  | R_ lobules Ⅹ | 22 | 23 | -32 | -41 | 21.51 |
|  | R_ lobules Ⅸ | 99 | 9 | -46 | -59 | 33.12 |
|  | L_ lobules Ⅸ | 90 | -9 | -46 | -45 | 26.17 |
|  | R_ACC | 21 | 47 | 13 | -21 | 19.28 |

**Abbreviations:** L: left; R: right; MNI, Montreal Neurological Institute; MTG, middle temporal gyrus; STG, superior temporal gyrus; MTG: middle temporal gyrus; ITG: inferior temporal gyrus; ITG: inferior temporal gyrus; MOG: middle occipital gyrus; IOG, inferior occipital gyrus; SOG : superior occipital gyrus; ACC, anterior cingulum; SFG: superior frontal gyrus; IFG_orb: orbital part of the inferior frontal gyrus; IFG: inferior frontal gyrus; IFG-tri: triangular part of the inferior frontal gyrus.

**Table S2.** Post hoc Analysis of the Main Effect of Diagnosis in ALFF

| Regions | Groups | Median value | P |
| --- | --- | --- | --- |
| R_ ParaHippocampal | SCH | -0.260 | 0.000 |
|  | NC | -0.369 |  |
| R_ Hippocampus | SCH | -0.345 | 0.000 |
|  | NC | -0.477 |  |
| R_MTG | SCH | -0.078 | 0.000 |
|  | NC | -0.173 |  |
| R_ Calcarine | SCH | 0.004 | 0.000 |
|  | NC | 0.471 |  |
| L_ Calcarine | SCH | 0.050 | 0.000 |
|  | NC | 0.458 |  |
| R_IOG | SCH | -0.125 | 0.000 |
|  | NC | 0.102 |  |
| L_IOG | SCH | 0.065 | 0.000 |
|  | NC | 0.284 |  |
| R_MOG | SCH | 0.007 | 0.000 |
|  | NC | 0.277 |  |
| L_MOG | SCH | 0.121 | 0.000 |
|  | NC | 0.371 |  |
| R_SOG | SCH | -0.180 | 0.000 |
|  | NC | 0.009 |  |
| L_ Fusiform | SCH | -0.272 | 0.000 |
|  | NC | -0.085 |  |
| R_Lingual | SCH | -0.380 | 0.000 |
|  | NC | -0.152 |  |
| L_Lingual | SCH | 0.060 | 0.000 |
|  | NC | 0.526 |  |
| L_Cuneus | SCH | 0.311 | 0.00 |
|  | NC | 1.012 |  |
| R_Cuneus | SCH | 0.274 | 0.000 |
|  | NC | 1.143 |  |
| R_ lobules Ⅸ | SCH | -0.530 | 0.000 |
|  | NC | -0.655 |  |
| L_ lobules Ⅵ/Ⅴ | SCH | -0.218 | 0.000 |
|  | NC | 0.030 |  |
| R_ lobules Ⅵ | SCH | -0.058 | 0.000 |
|  | NC | 0.366 |  |
| L_ Precuneus | SCH | -0.089 | 0.001 |
|  | NC | 0.044 |  |
| L_ Paracentral gyrus | SCH | -0.313 | 0.000 |
|  | NC | -0.184 |  |
| L_ postcentral gyrus | SCH | -0.154 | 0.000 |
|  | NC | 0.094 |  |

**Abbreviations:** L: left; R: right; MNI, Montreal Neurological Institute; MTG, middle temporal gyrus; STG, superior temporal gyrus; MTG: middle temporal gyrus; ITG: inferior temporal gyrus; ITG: inferior temporal gyrus; MOG: middle occipital gyrus; IOG, inferior occipital gyrus; SOG : superior occipital gyrus; ACC, anterior cingulum; SFG: superior frontal gyrus; IFG_orb: orbital part of the inferior frontal gyrus; IFG: inferior frontal gyrus; IFG-tri: triangular part of the inferior frontal gyrus.

**Table S3.** Post hoc Analysis of the Main Effect of Diagnosis in dALFF

| Regions | Groups | Median value | P |
| --- | --- | --- | --- |
| R_ ParaHippocampal | SCH | 0.226 | 0.000 |
|  | NC | 0.203 |  |
| R_ Hippocampus | SCH | 0.213 | 0.020 |
|  | NC | 0.203 |  |
| R_STG_Pole | SCH | 0.297 | 0.050 |
|  | NC | 0.285 |  |
| R_MTG_Pole | SCH | 0.297 | 0.050 |
|  | NC | 0.285 |  |
| R_Medial Frontal gyrus | SCH | 0.216 | 0.348 |
|  | NC | 0.214 |  |
| R_ACC | SCH | 0.305 | 0.003 |
|  | NC | 0.273 |  |
| R_Lingual | SCH | 0.260 | 0.000 |
|  | NC | 0.310 |  |
| L_Lingual | SCH | 0.331 | 0.000 |
|  | NC | 0.404 |  |
| R_ Calcarine | SCH | 0.283 | 0.000 |
|  | NC | 0.366 |  |
| L_ Calcarine | SCH | 0.327 | 0.000 |
|  | NC | 0.435 |  |
| L_Cuneus | SCH | 0.355 | 0.000 |
|  | NC | 0.490 |  |
| R_Cuneus | SCH | 0.370 | 0.000 |
|  | NC | 0.505 |  |
| R_IOG | SCH | 0.290 | 0.000 |
|  | NC | 0.350 |  |
| R_MOG | SCH | 0.291 | 0.000 |
|  | NC | 0.323 |  |
| R_ Fusiform | SCH | 0.291 | 0.000 |
|  | NC | 0.323 |  |
| R_ lobules Ⅷ | SCH | 0.227 | 0.002 |
|  | NC | 0.203 |  |
| R_ lobules Ⅹ | SCH | 0.226 | 0.005 |
|  | NC | 0.199 |  |
| R_ lobules Ⅸ | SCH | 0.270 | 0.001 |
|  | NC | 0.240 |  |
| L_ lobules Ⅸ | SCH | 0.305 | 0.003 |
|  | NC | 0.273 |  |

**Abbreviations:** L: left; R: right; MNI, Montreal Neurological Institute; MTG, middle temporal gyrus; STG, superior temporal gyrus; MTG: middle temporal gyrus; ITG: inferior temporal gyrus; ITG: inferior temporal gyrus; MOG: middle occipital gyrus; IOG, inferior occipital gyrus; SOG : superior occipital gyrus; ACC, anterior cingulum; SFG: superior frontal gyrus; IFG_orb: orbital part of the inferior frontal gyrus; IFG: inferior frontal gyrus; IFG-tri: triangular part of the inferior frontal gyrus.

**Table S4.** Brain Regions with Significant Group Differences in The Main Effect of Age

| Group differences | Regions | Cluster size  (voxels) | Peak MNI coordinate | | | Peak F  values |
| --- | --- | --- | --- | --- | --- | --- |
|  |  |  | X | Y | Z |  |
| *ALFF* | R_ Calcarine | 46 | 9 | -69 | 15 | 22.78 |
|  | L_IOG | 61 | -39 | -75 | -6 | 30.59 |
|  | L_MOG | 18 | -48 | -78 | 9 | 22.26 |
|  | L_SOG | 21 | -18 | -75 | 33 | 23.33 |
|  | R_MOG | 35 | 27 | -96 | 12 | 21.52 |
|  | R_Paracentral | 24 | 3 | -33 | 75 | 20.90 |
| *dALFF* | L_Fusiform | 21 | -33 | -9 | -27 | 14.45 |
|  | R_Pallidum | 21 | 17 | -2 | -7 | 15.90 |
|  | L_SFG | 37 | -21 | 18 | -24 | 14.94 |
|  | L_MFG | 60 | -26 | 1 | 56 | 21.98 |
|  | L_IFG_orb | 25 | -17 | 10 | -18 | 17.52 |
|  | L_IFG | 22 |  |  |  |  |
|  | L_STG_pole | 30 | -21 | 12 | -27 | 21.56 |

**Abbreviations:** L: left; R: right; MNI, Montreal Neurological Institute; IOG, inferior occipital gyrus; MOG, middle occipital gyrus; SOG: superior occipital gyrus; IFG_orb: orbital part of the inferior frontal gyrus; SFG: superior frontal gyrus; MFG, middle frontal gyrus; ITG, inferior temporal gyrus.

**Table S5.** Post hoc Analysis of the Main Effect of Age in ALFF

| Regions | Groups | Median value | P |
| --- | --- | --- | --- |
| L_IOG | Adult | -0.360 | 0.000 |
|  | Adolescence | -0.151 |  |
| L_MOG | Adult | -0.024 | 0.000 |
|  | Adolescence | 0.259 |  |
| L_SOG | Adult | -0.243 | 0.000 |
|  | Adolescence | -0.127 |  |
| R_Paracental | Adult | 0.501 | 0.000 |
|  | Adolescence | 0.827 |  |
| R_Calcarine | Adult | 0.102 | 0.000 |
|  | Adolescence | 0.537 |  |
| R_MOG | Adult | -0.024 | 0.000 |
|  | Adolescence | 0.259 |  |

**Abbreviations:** L: left; R: right; MNI, Montreal Neurological Institute; MFG, middle frontal gyrus; SFG: superior frontal gyrus; IFG_orb, orbital part of inferior frontal gyrus; IOG, inferior occipital gyrus; MOG, middle occipital gyrus.

**Table S6.** Post hoc Analysis of the Main Effect of Age in dALFF

| Regions | Groups | Median value | P |
| --- | --- | --- | --- |
| L_Fusiform | Adult | 0.205 | 0.000 |
|  | Adolescence | 0.174 |  |
| R_Pallidum | Adult | 0.184 | 0.000 |
|  | Adolescence | 0.161 |  |
| L_SFG | Adult | 0.174 | 0.000 |
|  | Adolescence | 0.159 |  |
| L_MFG | Adult | 0.256 | 0.000 |
|  | Adolescence | 0.218 |  |
| L_IFG_orb | Adult | 0.199 | 0.000 |
|  | Adolescence | 0.173 |  |
| L_IFG | Adult | 0.205 | 0.000 |
|  | Adolescence | 0.183 |  |
| L_STG_pole | Adult | 0.205 | 0.000 |
|  | Adolescence | 0.183 |  |

**Abbreviations:** L: left; R: right; MNI, Montreal Neurological Institute; MFG, middle frontal gyrus; SFG: superior frontal gyrus; IFG_orb, orbital part of inferior frontal gyrus; IOG, inferior occipital gyrus; MOG, middle occipital gyrus.

**Table S7.** Brain Regions with Significant Group Differences in the Interaction Effect

| Group differences | Regions | Cluster size  (voxels) | Peak MNI coordinate | | | Peak F  values |
| --- | --- | --- | --- | --- | --- | --- |
|  |  |  | X | Y | Z |  |
| *30TR_60% overlap* | L_IFG-tri | 27 | -50 | 24 | 18 | 17.43 |
| *50TR_80% overlap* | L_IFG-tri | 20 | -48 | 21 | 27 | 17.58 |

**Abbreviations:** L_IFG-tri, triangular part of the inferior frontal gyrus; MNI, Montreal Neurological Institute.

**Table S8.** Brain Regions with Significant Group Differences in the Main Effect of Diagnosis

| Group differences | Regions | Cluster size  (voxels) | Peak MNI coordinate | | | Peak F  values |
| --- | --- | --- | --- | --- | --- | --- |
|  |  |  | X | Y | Z |  |
| *30TR_60% overlap* | R_ParaHippocampal | 117 | 22 | -31 | -11 | 27.01 |
|  | R_Hippocampus | 81 | 27 | -10 | -21 | 18.18 |
|  | R_MTG | 30 | 48 | 6 | -27 | 12.54 |
|  | R_STG-Pole | 37 | 48 | 14 | -21 | 18.37 |
|  | R_MTG-Pole | 27 | 45 | 12 | -20 | 13.47 |
|  | L_Frontal Lobe (Medial) | 67 | 4 | 14 | -18 | 22.57 |
|  | L_Calcarine | 122 | -8 | -78 | 6 | 15.57 |
|  | R_Fusiform | 40 | 24 | -30 | -18 | 21.00 |
|  | R_Lingual | 167 | -10 | -61 | 1 | 26.22 |
|  | L_Lingual | 84 | -11 | -60 | 0 | 27.00 |
|  | R_IOG | 125 | 37 | -85 | -3 | 24.28 |
|  | L_IOG | 30 | -27 | -96 | -10 | 20.24 |
|  | R_MOG | 24 | 31 | -89 | 1 | 13.22 |
|  | L_Cuneus | 112 | -1 | -82 | 21 | 34.30 |
|  | R_Cuneus | 94 | 6 | -84 | 24 | 25.14 |
|  | R_Cerebelum Ⅸ | 116 | 12 | -43 | -45 | 29.12 |
|  | L_Cerebelum Ⅸ | 81 | -12 | -45 | -46 | 21.00 |
|  | R_Cerebelum Ⅷ | 32 | 20 | -43 | -50 | 16.80 |
|  | R_Cerebelum Ⅹ | 24 | 23 | -32 | -42 | 21.00 |
|  | R_Precuneus | 43 | 6 | -69 | 30 | 11.19 |
|  | L_Paracentral | 65 | -8 | -34 | 73 | 21.39 |
| *50TR_80% overlap* | R_ParaHippocampal | 112 | 27 | -14 | -24 | 23.02 |
|  | R_Hippocampus | 73 | 29 | -9 | -18 | 19.80 |
|  | R_MTG | 21 | 49 | 5 | -26 | 18.13 |
|  | R_STG-Pole | 37 | 48 | 13 | -21 | 17.35 |
|  | R_MTG-Pole | 24 | 48 | 10 | -26 | 14.82 |
|  | R_Medial Frontal gyrus | 45 | 8 | 52 | 9 | 16.77 |
|  | R_IFG-tri | 45 | -42 | 21 | 5 | 10.52 |
|  | R_ACC | 24 | 14 | 40 | 5 | 17.79 |
|  | R_Fusiform | 27 | 27 | -29 | -18 | 22.11 |
|  | L_Fusiform | -27 | -27 | -27 | -27 | 19.83 |
|  | R_Calcarine | 117 | 16 | -66 | 7 | 19.95 |
|  | L_Calcarine | 79 | -7 | -78 | 9 | 16.73 |
|  | L_Lingual | 29 | -10 | -62 | 0 | 20.87 |
|  | R_Lingual | 112 | 17 | -62 | -1 | 21.43 |
|  | R_IOG | 82 | 37 | -84 | -5 | 20.72 |
|  | L_Cuneus | 84 | -5 | -81 | 25 | 25.90 |
|  | R_Cuneus | 47 | 3 | -80 | 25 | 25.90 |
|  | L_Insula | 34 | -39 | 7 | 8 | 18.14 |
|  | R_ lobules Ⅸ | 100 | 16 | -38 | -49 | 22.57 |
|  | L_ lobules Ⅸ | 86 | -9 | -45 | -46 | 23.12 |
|  | R_ lobules 8 | 29 | 16 | -46 | -56 | 21.27 |
|  | R_ lobules Ⅵ/Ⅴ | 23 | 21 | -30 | -24 | 19.53 |
|  | R_ lobules Ⅹ | 21 | 23 | -30 | -41 | 25.81 |
| *80TR_60% overlap* | R_ lobules Ⅸ | 41 | 8 | -48 | -41 | 18.26 |
|  | L_ lobules Ⅸ | 41 | -9 | -45 | -46 | 18.16 |
|  | R_ParaHippocampal | 21 | 27 | 6 | -30 | 17.41 |
|  | R_STG | 20 | 28 | 6 | -23 | 13.72 |
|  | R_Lingual | 40 | 10 | -74 | -4 | 18.46 |

**Abbreviations:** L: left; R: right; MNI, Montreal Neurological Institute; ITG: inferior temporal gyrus; MTG, middle temporal gyrus; STG, superior temporal gyrus; MTG: middle temporal gyrus; ITG: inferior temporal gyrus; IFG-tri: triangular part of the inferior frontal gyrus; IOG, inferior occipital gyrus; SFG: superior frontal gyrus; MOG: middle occipital gyrus; ACC, anterior cingulum; IFG_orb: orbital part of inferior frontal gyrus; IFG-oper: opercular part of the inferior frontal gyrus.

**Table S9.** Brain Regions with Significant Group Differences in the Main Effect of Age

| Group differences | Regions | Cluster size  (voxels) | Peak MNI coordinate | | | Peak T  values |
| --- | --- | --- | --- | --- | --- | --- |
|  |  |  | X | Y | Z |  |
| *30TR_60% overlap* | L_Fusiform | 72 | -34 | -36 | -23 | 19.33 |
|  | L_Pallidum | 24 | -16 | -2 | -5 | 16.43 |
|  | R_Pallidum | 23 | 17 | -1 | -5 | 19.01 |
|  | R_Putamen | 28 | 36 | -4 | -1 | 16.55 |
|  | L_MFG | 117 | -36 | 18 | 40 | 19.64 |
|  | L_STG_Pole | 30 | -21 | 15 | -26 | 25.20 |
|  | L_IFG | 51 | -24 | 2 | 58 | 19.64 |
|  | L_SFG | 53 | -15 | 10 | 50 | 15.51 |
|  | L_IFG_orb | 46 | -21 | 16 | -23 | 16.10 |
|  | R_IFG_orb | 23 | 22 | 16 | -25 | 19.85 |
|  | L_Supp_Motor_Area | 27 | -12 | 1 | 64 | 15.90 |
|  | L_ParaHippocampal | 26 | -21 | -4 | -34 | 17.81 |
|  | L_Hippocampal | 37 | -21 | -8 | -12 | 16.53 |
|  | R_Hippocampal | 46 | 34 | -24 | -10 | 18.00 |
|  | L_ lobules Ⅳ/Ⅴ | 23 | -24 | -32 | -31 | 15.90 |
|  | R_Insula | 28 | 38 | 0 | 1 | 12.00 |
| *50TR_80% overlap* | L_Fusiform | 24 | -34 | -9 | -27 | 16.90 |
|  | L_Pallidum | 23 | -16 | -2 | -3 | 15.90 |
|  | R_Pallidum | 22 | 18 | -2 | 0 | 16.90 |
|  | R_Putamen | 23 | 35 | -3 | -1 | 16.50 |
|  | L_STG_Pole | 30 | -21 | 10 | -28 | 22.62 |
|  | L_SFG | 65 | -15 | 10 | 51 | 17.26 |
|  | L_MFG | 43 | -26 | -1 | 55 | 22.90 |
|  | L_IFG_orb | 30 | -18 | 10 | -21 | 16.10 |
|  | L_ParaHippocampal | 24 | -20 | -3 | -34 | 17.70 |
|  | L_Hippocampal | 20 | -20 | -13 | -12 | 14.60 |
|  | R_Insula | 20 | 39 | -1 | -11 | 13.10 |

**Abbreviations:** L: left; R: right; MNI, Montreal Neurological Institute; MFG, middle frontal gyrus; SFG: superior frontal gyrus; IFG_orb, orbital part of inferior frontal gyrus; IOG, inferior occipital gyrus; MOG, middle occipital gyrus.


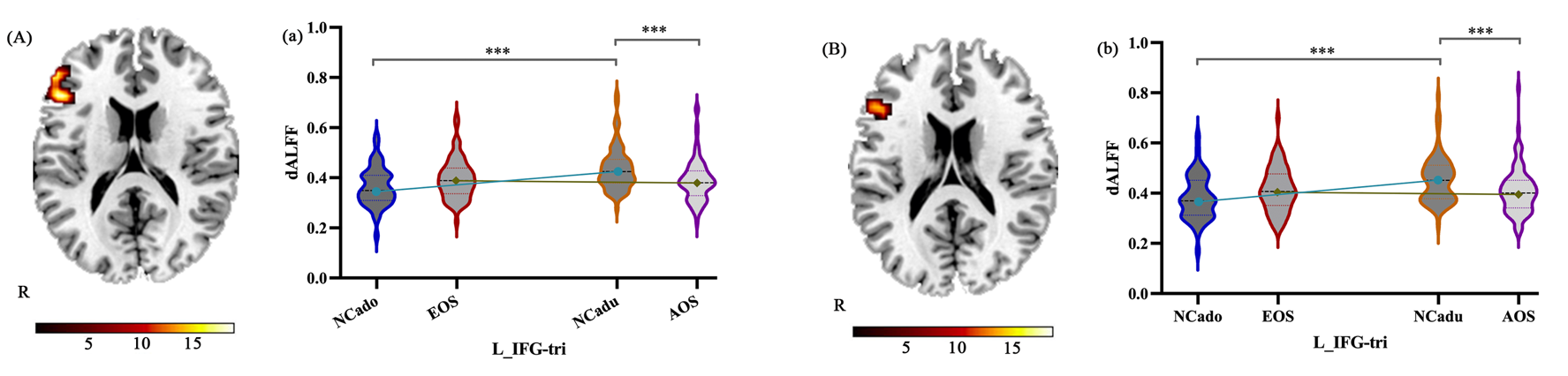


**Figure S1.** The main effect of age. (A) Dynamic ALFF with 30 TR and 60% overlap shows the significant main effect of age in using two-way ANOVA. (B)Dynamic ALFF with 50 TR and 80% overlap shows the significant main effect of age in using two-way ANOVA. (A-B) The statistical significance level was set at *P* < 0.001 (two-tail) and cluster size GRF corrected threshold of *P* < 0.05. EOS: first-episode early-onset schizophrenia patients; AOS: first-episode patients with adult-onset schizophrenia; NCadu: adult normal controls; NCado: adolescent normal controls; IFG-tri: triangular part of the inferior frontal gyrus; *:*P* < 0.05; **:*P*＜0.01; ***:*P*＜0.001


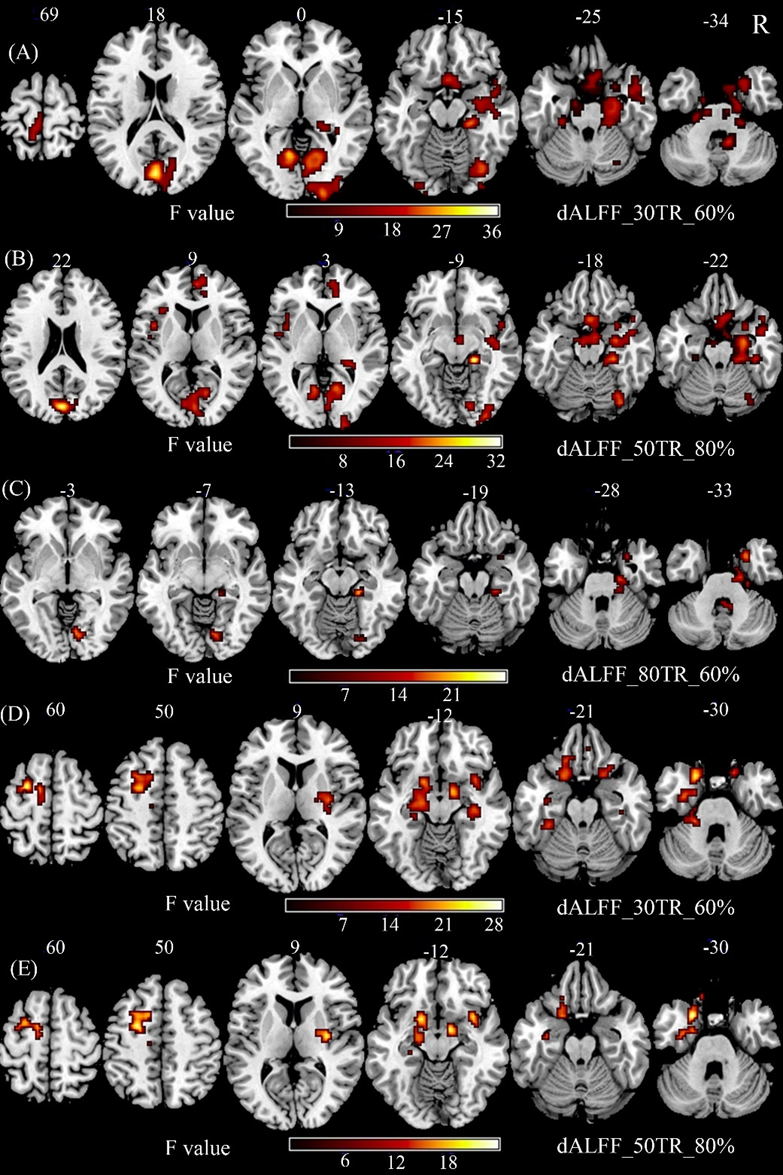


**Figure S2.** The main effect of diagnosis and age. (A) dALFF with 30 TR and 60% overlap shows the significant main effect of diagnosis in using two-way ANOVA. (B) dALFF with 50 TRs and 80% overlap shows the significant main effect of disease in using two-way ANOVA. (C)dALFF with 80 TRs and 60% overlap shows the significant main effect of disease in using two-way ANOVA. (D) dALFF with 30 TR and 60% overlap shows the significant main effect of age in using two-way ANOVA. (E) dALFF with 50 TR and 80% overlap shows the significant main effect of age in using two-way ANOVA. (E)The statistical significance level in figure 1(A-E) was set at *P* < 0.001 (two-tail) and cluster size at *P* < 0.05, GRF corrected.
